# Supplementary figures and images for: Schistosoma mansoni α-N-acetylgalactosaminidase (SmNAGAL) regulates coordinated parasite movement and egg production
Source: PLoS Pathog. 2022 Jan 13;18(1):e1009828. doi: 10.1371/journal.ppat.1009828 (PMC8791529; doi:10.1371/journal.ppat.1009828)

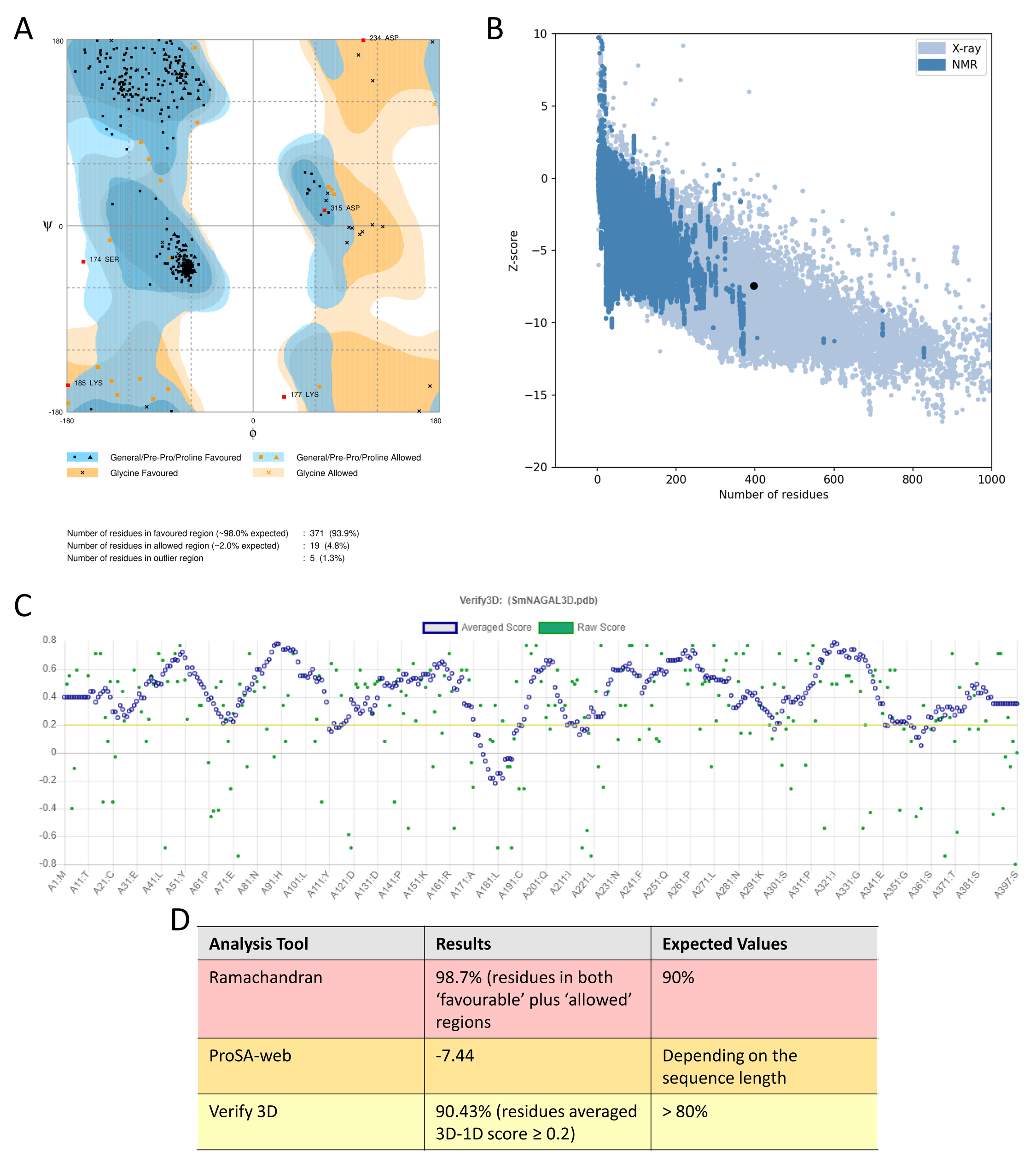

Supplement: S1 Fig — (A) The graphical representation of the RAMPAGE Ramachandran plot analysis for the Smp_089290 homology model. The plot depicts the torsional angles (phi, φ, x-axis and psi, Ψ, y-axis) of the amino acid residues in the homology model; this illustrates which combinations of angles for each atom is possible by considering their dimensions and van der Waals radii. Stable and unstable conformations of the model can, therefore, be plotted on the graph. (B) The graphical representation of the ProSA-web analysis for the Smp_089290 homology model. The z-score (-7.44, black dot) indicates the overall model quality, which is displayed in a plot that contains the z-scores of all experimentally determined protein chains in the current PDB database. The input structure is verified when it is found to be within the range of z-scores typically found for deposited proteins of similar size. (C) The graphical representation of the Verify 3D analysis for the Smp_089290 homology model. The analysis determines the compatibility of the atomic model (3D) of the input structure with its own amino acid sequence (1D) by assigning a structural class for each amino acid residue based on its location and environment (as part of an α-helix, β-strand or an interconnecting loop) and polarity. (D) A summary table of the analysis tool used (colour coded: RAMPAGE Ramachandran plot analysis = red, ProSA-web = orange and Verify 3D = yellow), the results obtained from the assessment of the Smp_089290 homology model and the expected values for verified structures. (TIF) [file ppat.1009828.s001.tif]

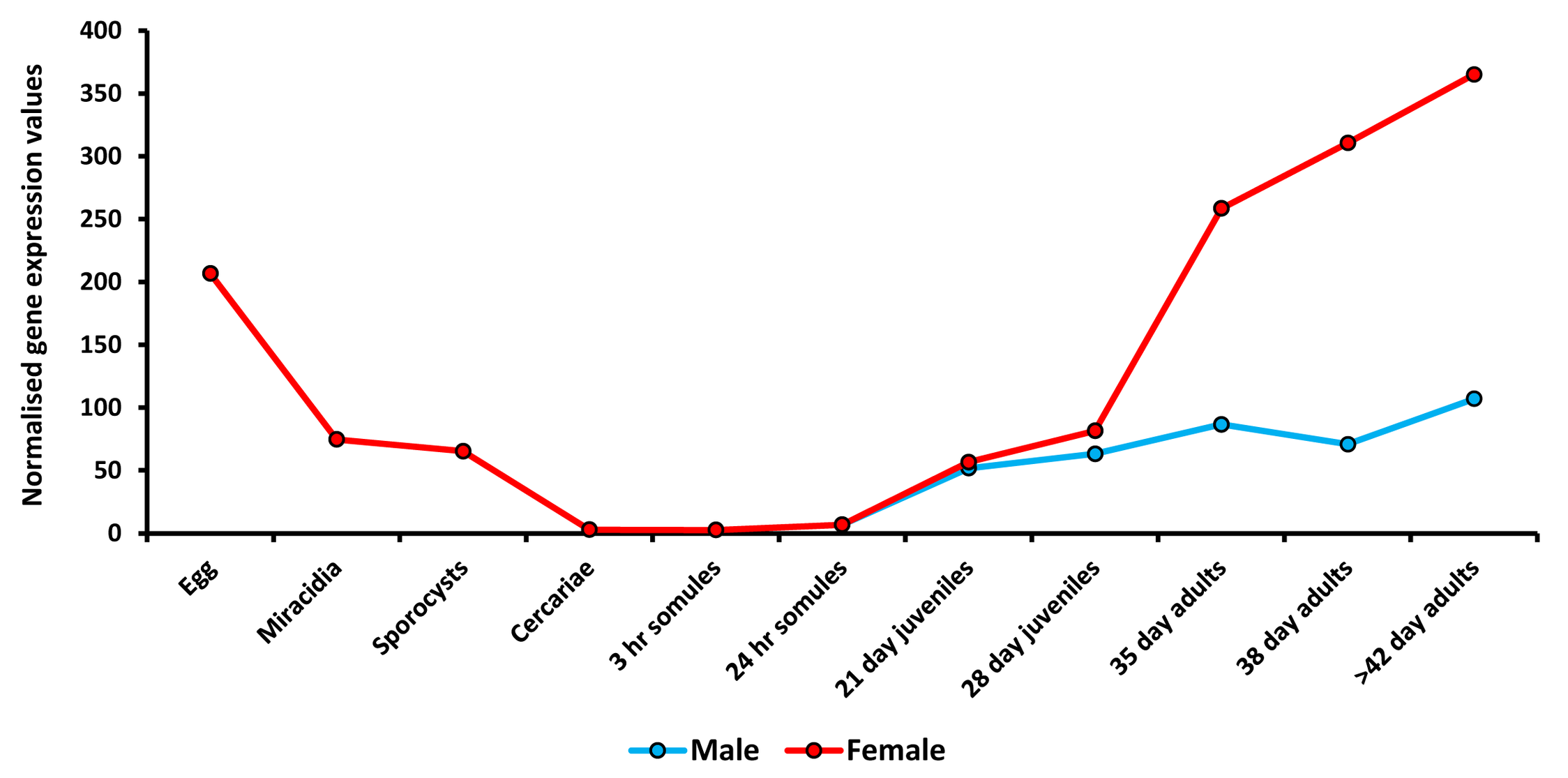

Supplement: S2 Fig — RNA-Seq meta data analysis of smp_089290 abundance for 11 lifecycle stages during mixed-sex infections. Individual expression values for male and female are only plotted for lifecycle stages where expression was assessed in sex-separated samples by Protasio et al. [51] and Lu et al. [52] (i.e. from ‘21 day juveniles’ to ‘>42 day adults’). A single expression value is plotted for lifecycle stages where expression was assessed in mixed-sex samples by Anderson et al. [48], Wang et al. [49] and Protasio et al. [50] (i.e. from ‘egg’ to ‘24 hr somules’). (TIF) [file ppat.1009828.s002.tif]

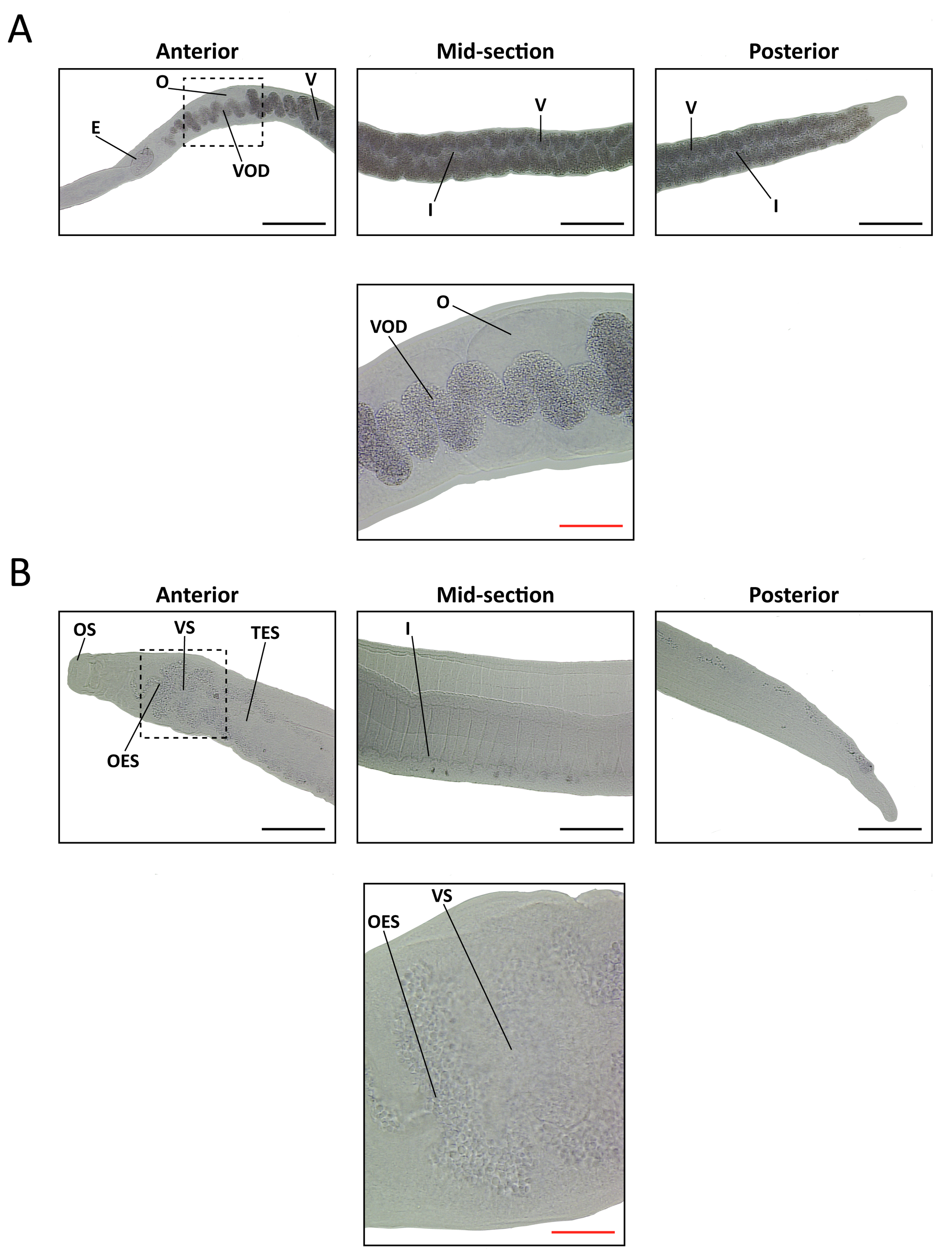

Supplement: S3 Fig — Images of the anterior, mid-section and posterior (10x magnification) of (A) female and (B) male schistosomes as well as anterior images with a higher magnification (40x magnification, area depicted by black dashed box). Structures labelled include egg (E), ovary (O), vitellarium (V), vitello-oviduct (VOD), intestine (I), oral sucker (OS), oesophagus (OES), ventral sucker (VS) and testes (TES). Black scale bars = 200 μm and red scale bars = 50 μm. (TIF) [file ppat.1009828.s003.tif]

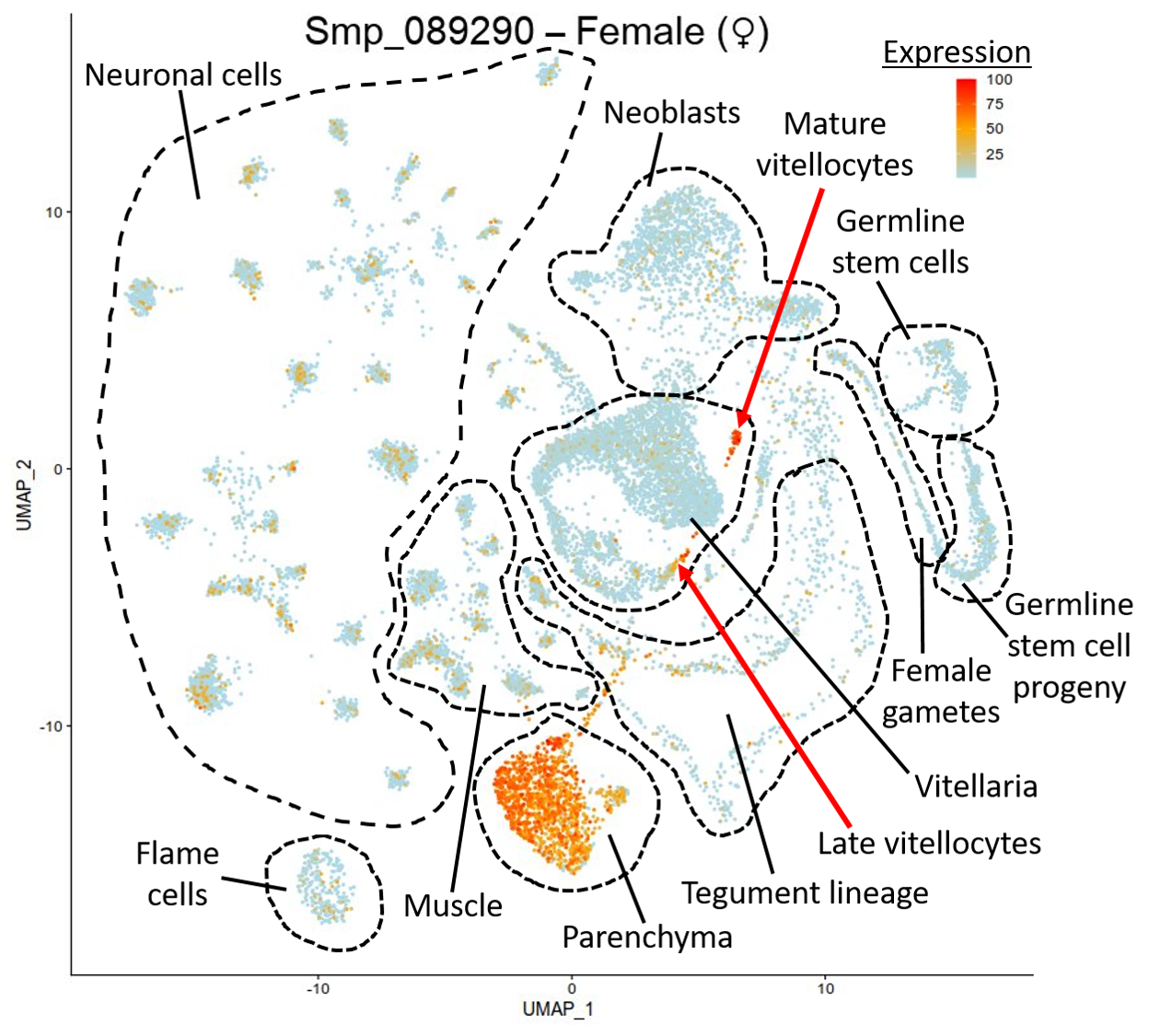

Supplement: S4 Fig — Labelled UMAP projection plot of various cell clusters highlighted by black dashed regions. smp_089290 scRNA-Seq expression values shown in this plot were generated from sexually mature adult female samples only. Expression values are normalised to a scale of 0–100 and colour coded (blue = low, red = high). Mature vitellocytes and late vitellocytes are labelled by red arrows. (TIF) [file ppat.1009828.s004.tif]

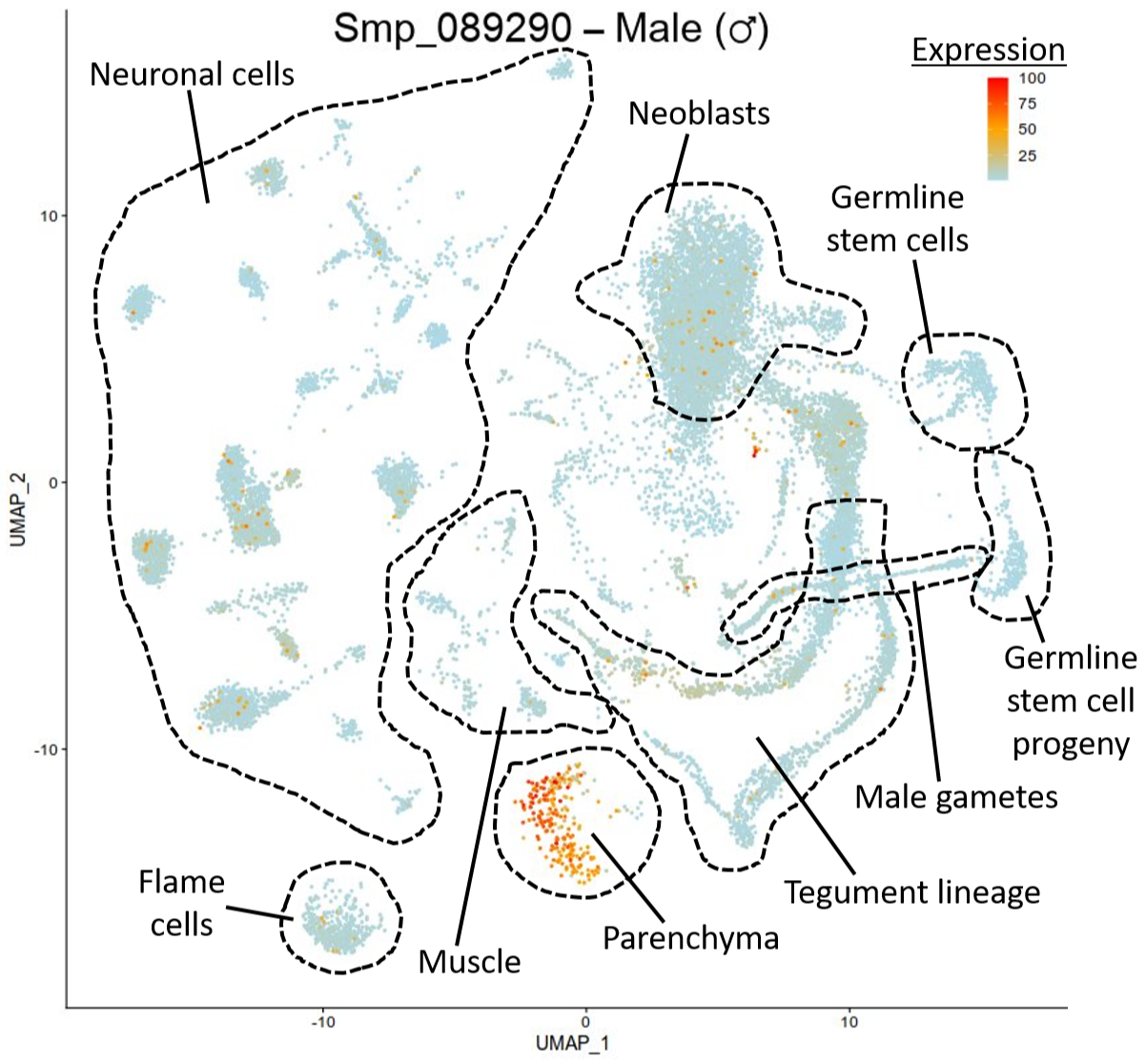

Supplement: S5 Fig — Labelled UMAP projection plot of various cell clusters highlighted by black dashed regions. smp_089290 scRNA-Seq transcript expression values shown in this plot were generated from adult male samples only. Expression values are normalised to a scale of 0–100 and colour coded (blue = low, red = high). (TIF) [file ppat.1009828.s005.tif]

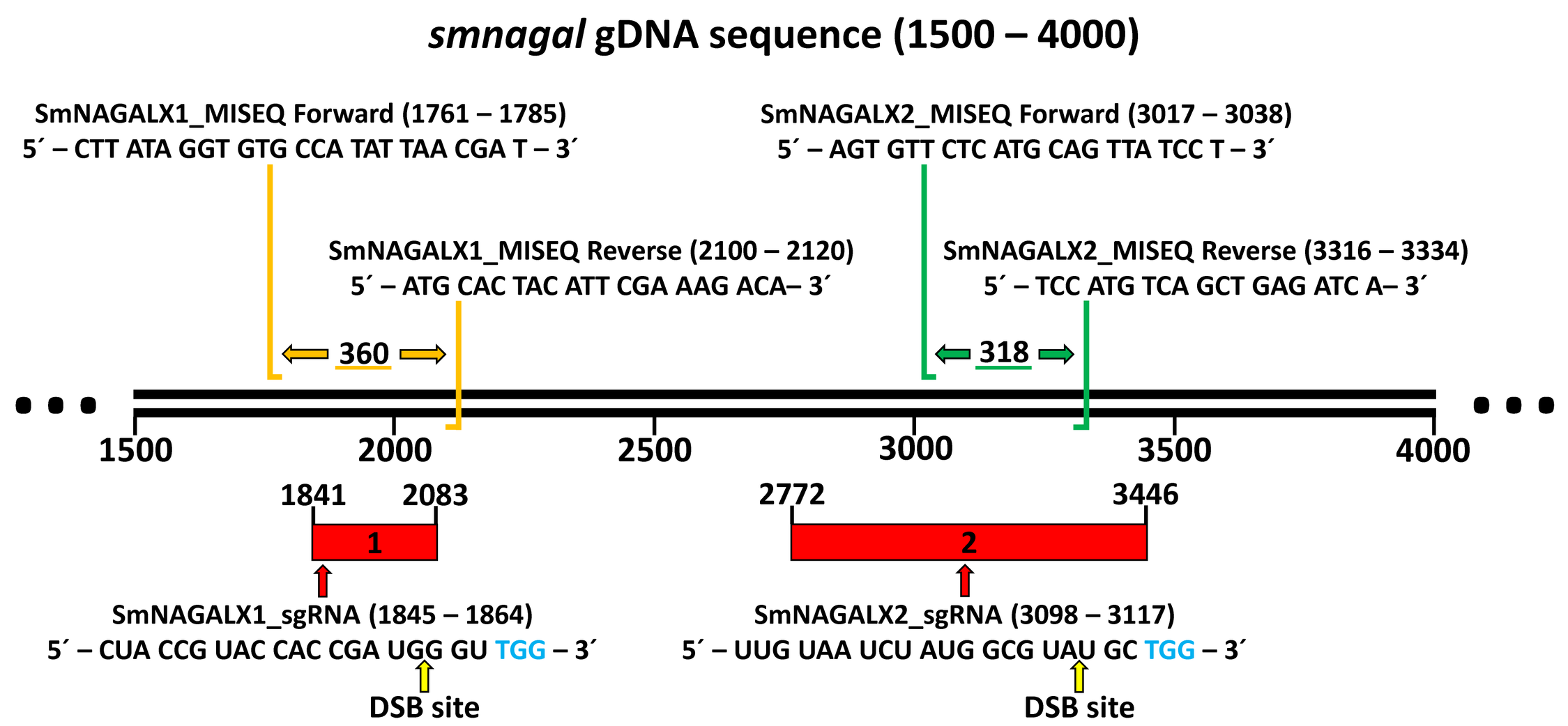

Supplement: S6 Fig — Diagrammatic representation of the genomic sequence of smnagal from nucleotide (nt) positions 1500–4000 (represented by double lines) based on WormBase ParaSite entry [27]. Exons are depicted as red boxes with nt positions indicated above 5´ and 3´ ends. Numbers written inside each exon represent their position in the gene sequence. Positions and nt sequences of SmNAGALX1_sgRNA and SmNAGALX2_sgRNA are shown below exon 1 and 2, respectively (represented by red arrows). DSB sites for SmNAGALX1_sgRNA and SmNAGALX2_sgRNA are indicated by yellow arrows, which are located three nts upstream of the PAM sequence highlighted in light blue (note: the PAM sequence is not part of the sgRNA sequence). Positions and nt sequences of SmNAGALX1_MISEQ (represented by orange lines) and SmNAGALX2_MISEQ (represented by green lines) primers are shown along the genomic sequence. Amplicon lengths of SmNAGALX1_MISEQ and SmNAGALX2_MISEQ products are underlined in orange and green, respectively. (TIF) [file ppat.1009828.s006.tif]

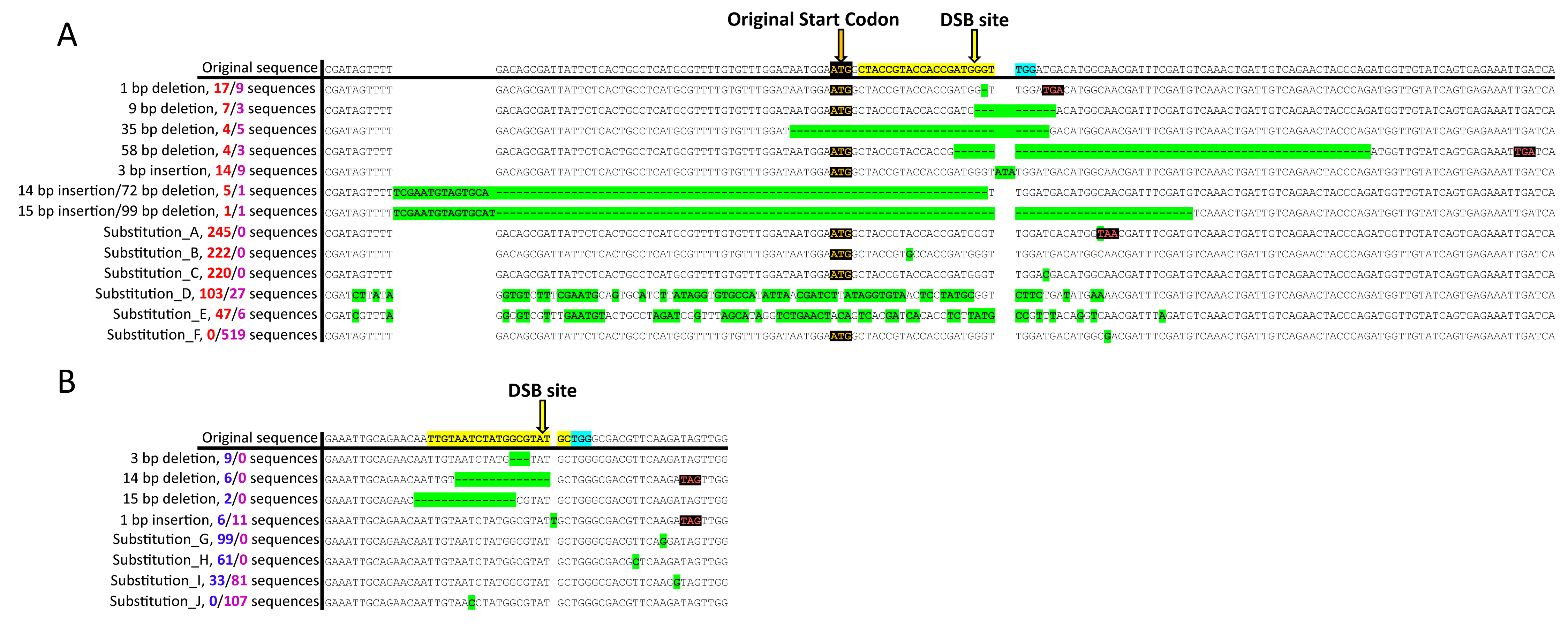

Supplement: S7 Fig — Multiple sequence alignment (MSA) showing all insertions, deletions, insertions with deletions and representative substitutions (i.e. supported by more than one sequence read) identified in modified sequence reads by CRISPResso2 analysis. Modified sequence reads are aligned with the original unmodified smnagal nucleotide (nt) sequence. Alignments are grouped as follows: (A) Experimental treatment groups targeting exon 1 and (B) Experimental treatment groups targeting exon 2. A colour code is used to show the number of sequence reads each indel/substitution appears in for SmNAGALX1 (red), SmNAGALX2 (dark blue) and dual SmNAGALX1/X2 plasmid treated worms (purple). Nts that are modified by each indel/substitution within the alignment are coloured green. Insertions appear as additional nts not found in the original sequence, deletions appear as lines (-) and substitutions appear as replaced nts located in the same positions as the original sequence. The nt sequences of SmNAGALX1_sgRNA and SmNAGALX2_sgRNA are highlighted in yellow within the original sequence. DSB sites for SmNAGALX1_sgRNA and SmNAGALX2_sgRNA are indicated by yellow arrows, which are located three nts upstream of the PAM sequence highlighted in light blue (note: the PAM sequence is outside and downstream of the sgRNA sequence). An additional colour code is used to distinguish between start (orange nts) and termination (red nts) codons found in the sequences, which have been highlighted in black. (TIF) [file ppat.1009828.s007.tif]

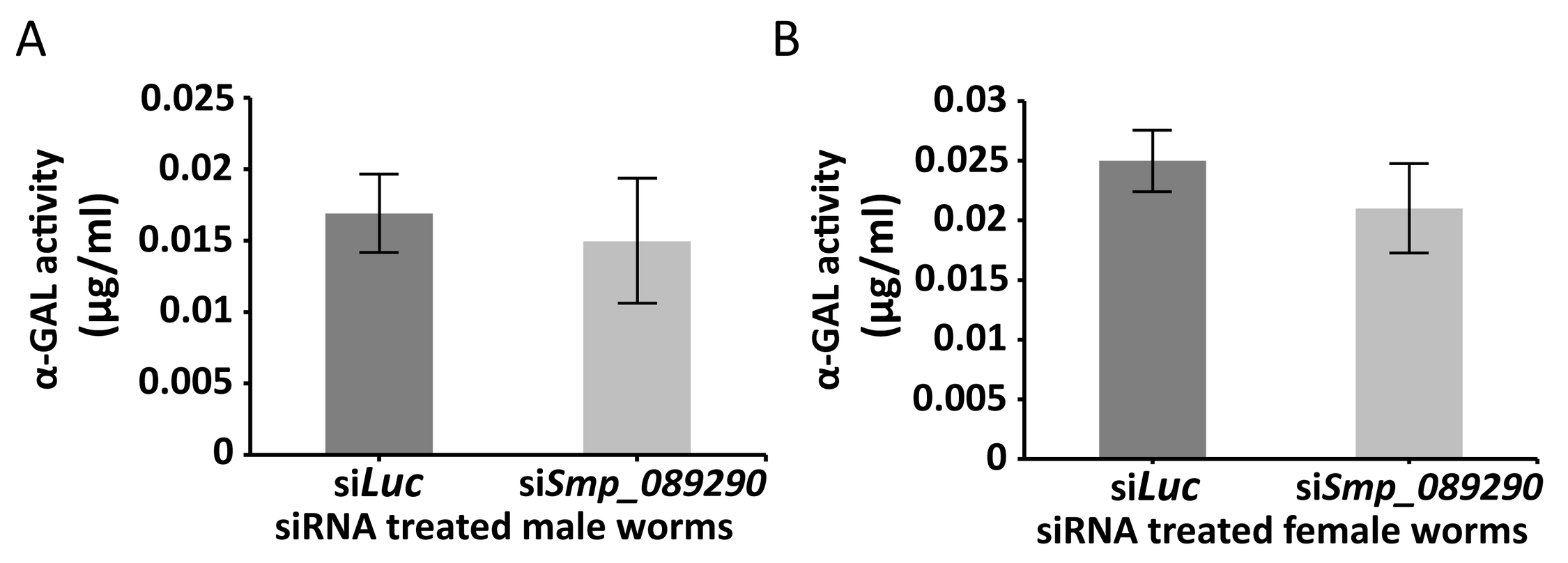

Supplement: S8 Fig — (A) 6.45 μg of siRNA treated adult male-derived SWAP and (B) 2.44 μg of siRNA treated adult female-derived SWAP were measured for α-GAL using α-GAL colorimetric substrates. Final absorbances were quantified at 410 nm. Using linear trendline equations generated from α-GAL standard curves, α-GAL activity (μg/ml) were calculated for each sample. No statistical significance in α-GAL activity between samples was observed (Student’s t-test, two tailed, unequal variance). (TIF) [file ppat.1009828.s008.tif]

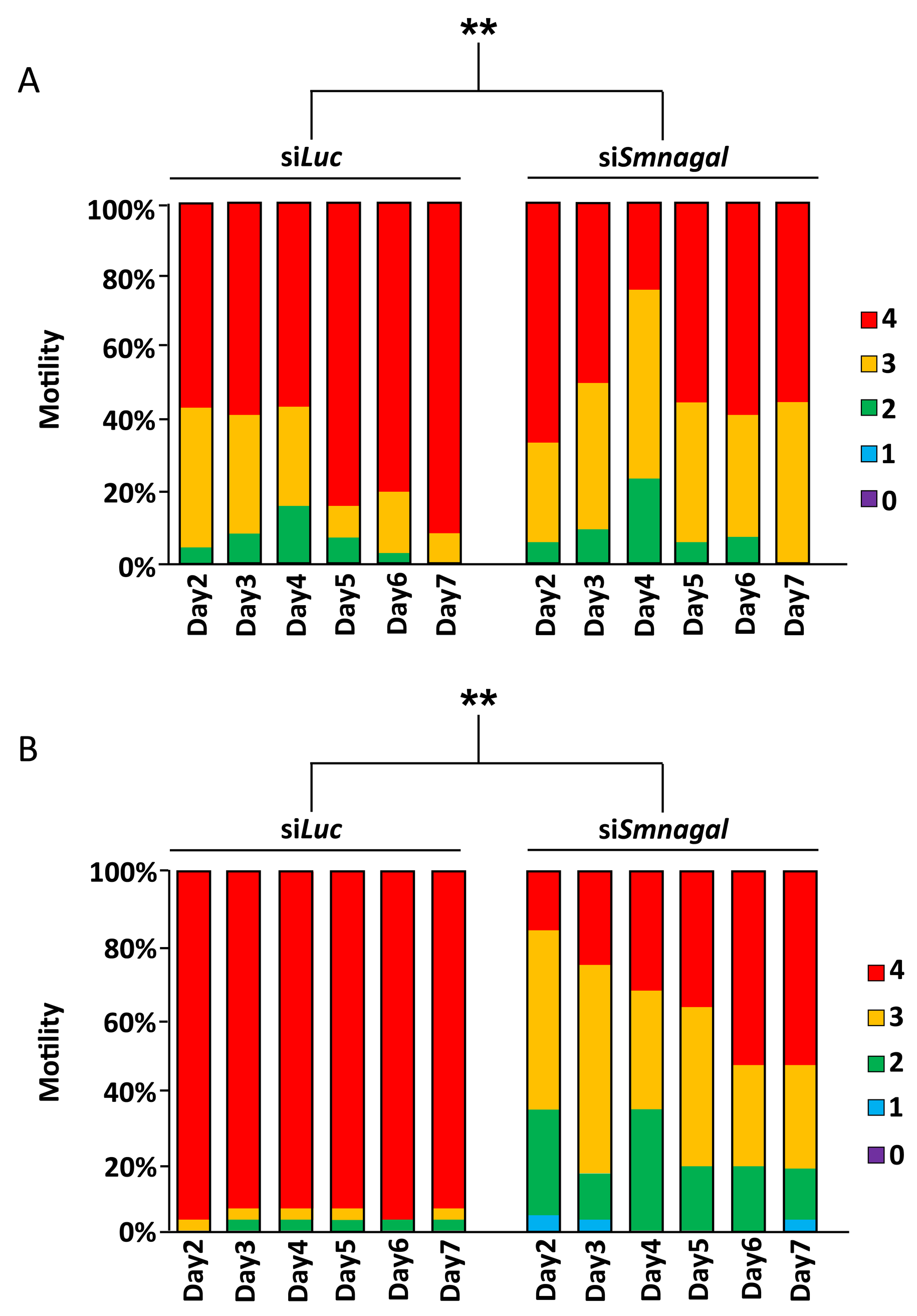

Supplement: S9 Fig — The motility of individual worms (five adult pairs per well) were scored between 4–0 based on the WHO-TDR scoring matrix guidelines; 4 = normal active/paired up, 3 = slowed activity, 2 = minimal activity and occasional movement of head and tail, 1 = absence of motility apart from gut movements and 0 = total absence of motility. The total occurrences of each score was plotted per day for siLuc treated and siSmNAGAL treated adult female (A) and male (B) worms starting from day two after electroporation up until day seven (day of electroporation considered as day zero). Statistical significance is indicated (General Linear Mixed-Effects Model, NLME and EMMEANS R packages, ** = p<0.01). Day one was not included in the statistical analysis due to worms appearing stunned and immobile, likely due to electroporation manipulation. Six wells/biological replicates per siRNA treatment were used for this analysis. (TIF) [file ppat.1009828.s009.tif]

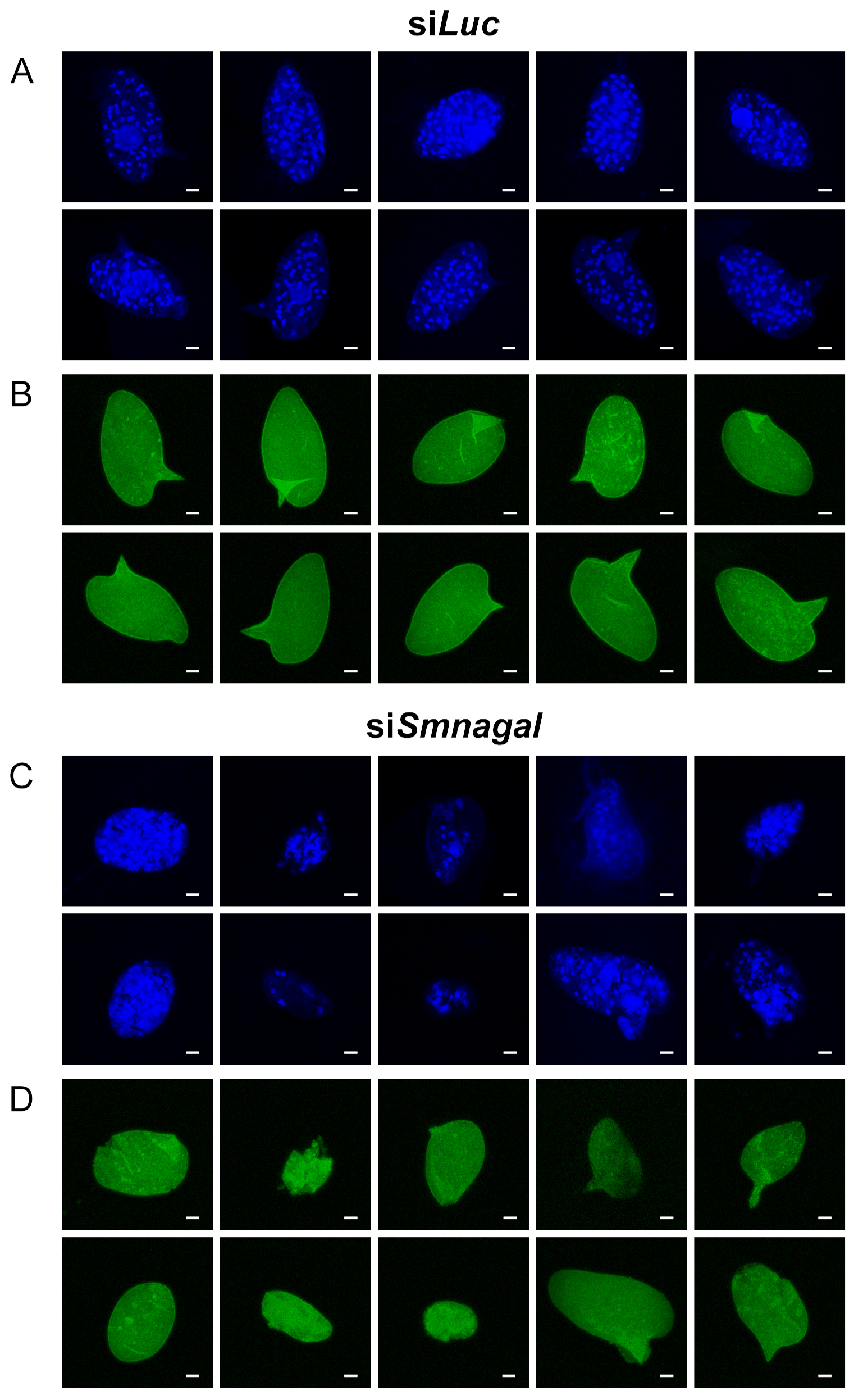

Supplement: S10 Fig — Images of eggs from siLuc treated worm pairs depicting (A) vitellocytes under the blue channel (i.e. excitation wavelength = 405 nm and emission wavelength = 461 nm) and (B) auto-fluorescence under the green channel (i.e. excitation wavelength = 488 nm and emission wavelength = 520 nm). Images of eggs from siSmNAGAL treated worm pairs depicting (C) vitellocytes under the blue channel and (D) auto-fluorescence under the green channel. Blue = DAPI+ cells, green = egg auto-fluorescence and white scale bars = 20 μm. (TIF) [file ppat.1009828.s010.tif]

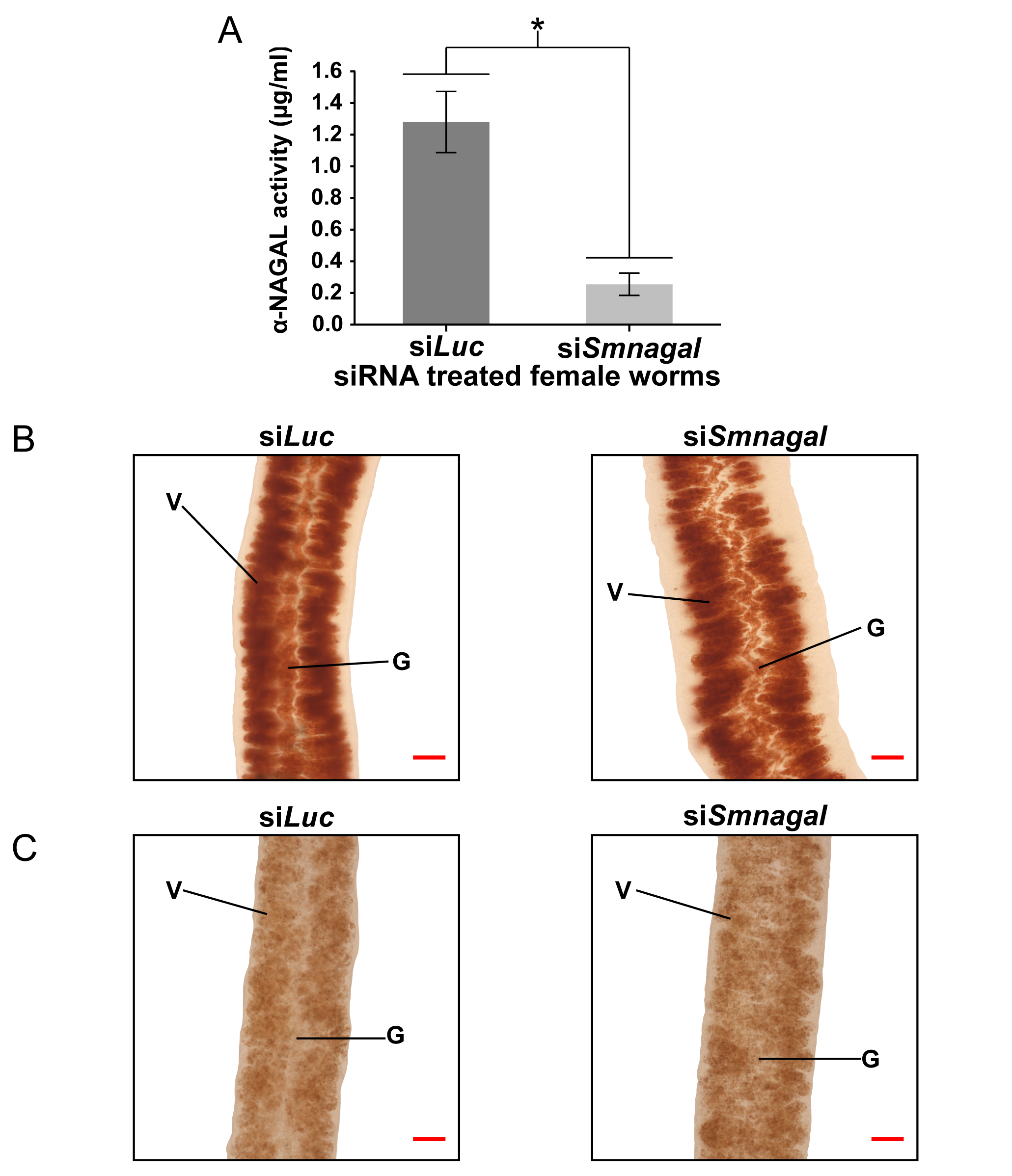

Supplement: S11 Fig — (A) α-NAGAL activity was measured in SWAP derived from siLuc treated and siSmnagal treated adult female worms (1.08 μg) as described above. Statistical significance is indicated (Student’s t-test, two tailed, unequal variance, * = p<0.05). Representative images of siRNA-treated adult female worms stained with (B) Fast Blue BB or subjected to (C) diphenol oxidase localisation (20x magnification, red scale bars = 50 μm). Structures labelled include vitellarium (V) and gut (G). (TIF) [file ppat.1009828.s011.tif]
