# Supplementary material for: Schistosoma mansoni α-N-acetylgalactosaminidase (SmNAGAL) regulates coordinated parasite movement and egg production
Source: PLoS Pathog. 2022 Jan 13;18(1):e1009828. doi: 10.1371/journal.ppat.1009828 (PMC8791529; doi:10.1371/journal.ppat.1009828)
Supplement: S1 Table — The number of sequence reads at each of the three initial processing stages before indel characterisations can be made by further CRISPResso2 analysis is presented. These three initial processing stages are “Reads in inputs” (highlighted in red, first stage), “Reads after pre-processing” (highlighted in blue, second stage) and “Reads aligned” (highlighted in yellow, third stage). “Reads in inputs” refers to the total number of sequence reads from raw MiSEQ sequencing data. “Reads after pre-processing” refers to the number of sequence reads after PCR amplification or trimming artefacts are removed. “Reads aligned” refers to the number of sequence reads that are of high quality (>60% homology to reference amplicon sequence), which are used for indel characterisations. The table also lists the primer pair set and sample (samples amplified by SmNAGALX1_MiSEQ and SmNAGALX2_MiSEQ primers are highlighted in orange and green, respectively) used for each barcoded MiSEQ amplicon library constructed. (DOCX) [file ppat.1009828.s014.docx]

**S1 Table. Initial processing of MiSEQ deep-coverage sequence reads for CRISPResso2 analysis.**

| **Primer pair set** | **Sample** | **Reads in inputs** | **Reads after pre-processing** | **Reads aligned** |
| --- | --- | --- | --- | --- |
| SmNAGALX1_MiSEQ primers (targeting exon 1) | Scramble | 588918 | 588918 | 139154 |
|  | SmNAGALX1 | 1469667 | 1048675 | 429175 |
|  | Dual SmNAGALX1/X2 | 1081902 | 535084 | 258576 |
| SmNAGALX2_MiSEQ primers (targeting exon 2) | Scramble | 722655 | 683478 | 176041 |
|  | SmNAGALX2 | 1916911 | 1916911 | 103559 |
|  | Dual SmNAGALX1/X2 | 1838265 | 1683634 | 136129 |
