# Supplementary material for: Schistosoma mansoni α-N-acetylgalactosaminidase (SmNAGAL) regulates coordinated parasite movement and egg production
Source: PLoS Pathog. 2022 Jan 13;18(1):e1009828. doi: 10.1371/journal.ppat.1009828 (PMC8791529; doi:10.1371/journal.ppat.1009828)
Supplement: S2 Table — The mutation frequencies attributable to genome editing (i.e. insertions, deletions, insertions with deletions, and substitutions) in smnagal-edited worms as quantified by CRISPResso2 analysis is indicated. The percentage of unmodified sequence reads is included. The primer pair set and sample (samples amplified by SmNAGALX1_MiSEQ and SmNAGALX2_MiSEQ primers are highlighted in orange and green, respectively) used for each barcoded MiSEQ amplicon library constructed are indicated. (DOCX) [file ppat.1009828.s015.docx]

**S2 Table. Detectable frequencies of insertions, deletions, insertions with deletions and substitutions in *smnagal*-edited worms as quantified by CRISPResso2 analysis.**

|  |  | **Mutation frequencies (%)** | | | | |
| --- | --- | --- | --- | --- | --- | --- |
| **Primer Pair set** | **Sample** | **Unmodified** | **Insertions** | **Deletions** | **Insertions with deletions** | **Substitutions** |
| SmNAGALX1_MiSEQ primers (targeting exon 1) | SmNAGALX1 | 99.7306 | 0.0032 | 0.0075 | 0.0014 | 0.2570 |
|  | Dual SmNAGALX1/X2 | 99.7400 | 0.0034 | 0.0073 | 0.0007 | 0.2506 |
| SmNAGALX2_MiSEQ primers (targeting exon 2) | SmNAGALX2 | 99.6900 | 0.0057 | 0.0164 | 0 | 0.2870 |
|  | Dual SmNAGALX1/X2 | 99.7517 | 0.0081 | 0 | 0 | 0.2402 |
