# Supplementary material for: Schistosoma mansoni α-N-acetylgalactosaminidase (SmNAGAL) regulates coordinated parasite movement and egg production
Source: PLoS Pathog. 2022 Jan 13;18(1):e1009828. doi: 10.1371/journal.ppat.1009828 (PMC8791529; doi:10.1371/journal.ppat.1009828)
Supplement: Attachment — Submitted filename: SmNAGAL Response to reviewers_Final.docx [file ppat.1009828.s016.docx]

**Point-by-point Response to the Reviewers’ Comments on PPATHOGENS-D-21-01500**

**We thank the reviewers very much for their detailed and constructive comments on our manuscript. Below, we provide a point-point-response (in green) to the Reviewers’ questions (in black).**

**Part II – Major Issues: Key Experiments Required for Acceptance**

Reviewer #1: No further experiments are required for the manuscript.

Reviewer #2: Lines 378 and 619-620: has this alpha-tubulin isoform (smat1) been tested and proven for its suitability as reference gene for qRT-PCR analyses and the determination of sex-specific differences? In case yes, provide a reference for this (or data, which confirm this), and provide the Smp_number of smat1. In case smat1 represents Smp_090120, this gene is not equally expressed between males and females, and there is a pairing influence on its transcript level (Lu et al. 2018). This may have influenced the results in Fig. 4, which compared sex-specific transcript patterns. Although this reviewer in principle agrees on the basis of numerous different results also from other groups that SmNAGAL is likely expressed with a female-bias, Smp_090120 is not well suited as a reference gene for this kind of analysis and may have corrupted results and statistics.

***smat1* does indeed represent Smp_090120. In our hands, the Ct value of *smat1* expression in males (16.35) was lower than that found for females (19.90). Therefore, we agree that its use as a reference gene for this qRT-PCR analysis is not ideal. We have subsequently repeated the qRT-PCR analysis using *S*. *mansoni* Ras-Related GTP-binding protein D (*smrgbd*; Smp_026090) and Tubulin-specific chaperone D (*smtscd*; Smp_153590) as reference genes. Using Normfinder (Andersen *et al* 2004 doi: 10.1158/0008-5472.CAN-04-0496), we determined that their expression across nine schistosome lifecycle stages (Lu and Berriman 2018 doi: 10.1101/308189) was low and stable. The Ct values of *smrgbd* (males = 22.34, females = 22.95) and *smtscd* (males = 22.93, females = 23.93) were shown to be more consistent between sexes than that of *smat1*. A geometric mean method analysis (Vandesompele *et al* 2002 doi: 10.1186/gb-2002-3-7-research0034) to quantify *smp_089290* transcript levels (compared to *smrgbd* and *smtscd*) in freshly perfused male and female schistosomes was performed as previously described (Cardoso *et al* 2021 doi: 10.1016/j.ijbiomac.2021.01.180). As expected (and previously reported in the originally submitted manuscript), *smp_089290* (*smnagal*) demonstrates a female-bias expression (shown below). This new data will now replace Figure 4C and the methodology has been updated to reflect the addition of this qRT-PCR analysis.**

Lines 687-690: have the authors checked whether the selected siRNAs against SmNAGAL were specific for this gene and failed targeting also one or more of the other closely related genes? It would be beneficial to show the transcript profiles of the related genes (see line 537 and Smp_334700) to convince the reader the KD was gene-specific for SmNAGAL only.

**We have checked that the selected siRNAs targeting *smnagal* were specific only to Smp_089290 and did not target the other closely related mRNAs (i.e. Smp_170840, Smp_179250, Smp_247760, Smp_247750 and Smp_334700). As shown in the alignment below, none of the closely related genes have significant homology towards the selected siRNA (underlined in Smp_089290; conserved nucleotides = green, non-conserved nucleotides = yellow). Smp_247750 has the highest number of nucleotides conserved (i.e. 13 out of 19) but 15 out of 19 nucleotides is considered to be the lower limit for a siRNA to successfully degrade a targeted mRNA sequences (Kim 2010, doi: https://doi.org/10.1007/978-1-60761-588-0_5, Fakhr *et al* 2016, doi:** **https://doi.org/10.1038/cgt.2016.4). Therefore, we believe the additional qRT-PCR analyses quantifying the other closely related genes is unnecessary.**

```
Smp_089290      GTAGATGATTGTTGGCCAGAATGGAAACGAGATTCTAGGACTAATGAAATCGTTGCAGAT	225
Smp_170840      ATTGATGACTTTTGGTTGGCAAACAAACGTGATTCGAAAATGAATAATATGACGGCCTAT	66
Smp_247750      CTTGATGATTGTTGGTTAGCGAAACAGCGTGATCCGAAGACGAATAGAATAATGGCAGAT	300
Smp_186710      ATTGATGATTGTTGGTTATCAAGACTGCGTGATCCGAAGACGGTTAGACTAATGCCAGAT	300
Smp_334700      ------------------------------------------------------------	0
Smp_179250      ATAGATGATTGTTGGTTATCAAGACTGCGTGATACGAAGACGGTTGCACTATTGCCAGAT	300
Smp_247760      ATAGATGATTGTTGGTTATCAAGACTGCGTGATACGAAGACGGTTGCACTATTGCCAGAT	300
```

Lines 906-913: the authors speculate that SmNAGAL may affect vitellocyte development. They assume that the normal development from immature, undifferentiated vitellocytes to mature, fully differentiated vitellocytes is interrupted in knock-down/knock-out females and does not proceed from stage 1 on. Although this seems plausible on the first view, no data have been provided substantiating this idea. The authors may envisage to stain worms (e.g. by lipid staining) to visualize this effect by bright field microscopy (see protocols of the Collins lab) or to perform confocal microscopy to investigate the loss/reduction of mature vitellocytes in KD or KO worms. The authors also speculate about fewer mature vitellocytes containing functionally active tyrosinases in KD or KO worms. In the past the same group has worked in this field and it may be easy for them to substantiate this idea by tyrosinase assays on tissue sections and/or qRT-PCR analyses in KD or KO worms.

**We have performed additional staining experiments on siRNA treated-adult female worms to visualise how *smnagal* knockdown affects vitellocyte development and tyrosinase activity using bright-field microscopy. For vitellocyte development, we have stained female worms with Fast Blue BB (Wang *et al* 2019, doi: https://doi.org/10.1371/journal.pbio.3000254) to visualise the vitelline droplets within mature vitellocytes. For visualisation of tyrosinase activity within mature vitellocytes, we have performed diphenol oxidase localization assays on female worms (Fitzpatrick *et al* 2007, doi: https://doi.org/10.1096/fj.06-7314com). We observed no difference between siRNA treatments (siLuc vs siSmNAGAL) for both staining methods despite significant reductions in α-NAGAL enzymatic activity (see new figure below). This has led us to revise our initial interpretation of how SmNAGAL influences egg production, which has been included in the discussion and is also indicated below:**

**‘Here, diminished numbers of eggs produced by *smnagal* depleted adults exhibited two predominant abnormalities; lack of typical vitellocyte structuring and spacing (Fig 7E, S11 and S12 Figs) and reductions in egg volume (Fig 7D). *smnagal* deficiency, however, does not influence vitellocyte incorporation as DAPI^+^ cells are observed in all eggs obtained from si*SmNAGAL* treatment groups. Reductions in SmNAGAL enzymatic activity (S13A Fig) also does not appear to influence the presence of vitelline droplets (S13B Fig) or the activity of tyrosinase (S13C Fig) in mature vitellocytes (within the vitellaria). While both of these features (along with lipid droplets) contribute to the formation of hardened/tanned eggs [75, 78-82], our data would suggest that their presence is unaffected by *smnagal* knockdown. Instead, SmNAGAL activity seems to affect an area of egg-laying biology (involving the vitellaria and vitellogenesis) that is currently unknown, but is associated with normal spacing of mature vitellocytes within the *in vitro* laid eggs (IVLEs) and total egg volume (i.e. si*SmNAGAL* treatment groups - approximately 15,000 µm^3^ per egg; si*Luc* treatment groups - approximately 35,000 µm^3^ per egg; Fig 7D). In some cases, *smnagal* deficiency also contributed to additional observed phenotypes such as abnormal shaped eggs and incomplete development of the lateral spine (S12 Fig). Therefore, in addition to adult worm motility, SmNAGAL clearly participates in vitelline droplet/tyrosinase-independent mechanisms of oviposition.’**

The conclusion that inhibition of SmNAGAL may lead to the development of novel anthelmintics is premature at this stage of the functional characterization of this gene.

**We agree that this statement is premature and, therefore, have edited this sentence. This new sentence has been added to our manuscript and can be read below:**

**‘Further characterisation of SmNAGAL and other functionally important glycosyl hydrolases may lead to the development of a novel anthelmintic class of compounds.’**

**Part III – Minor Issues: Editorial and Data Presentation Modifications**

Reviewer #1: Page 6 Line 121 : "Within GH family 27 are the related lysosomal enzymes ..." This sentence needs to be re-written.

**As requested, we have re-written this sentence. This new sentence has been added to our manuscript and can be read below:**

**‘GH family 27 comprises mainly α-galactosidases (α-GAL) and α-N-acetylgalactosaminidases (α-NAGAL) responsible for the cleavage of terminal α-D-galactose and α-N-acetylgalactosamine residues from glycosylated substrates, respectively.’**

Reviewer #2: Line 256: 1,463 bp

**We have added the comma as requested.**

Line 409/411: is there a reference for the relaxation protocol? Why is killing with MgCl2 done ahead of fixation in formaldehyde?

**We have added the reference for the relaxation protocol into the manuscript as requested (Collins et al 2013, doi: 10.1038/nature11924). In the protocol being followed, MgCl_2_ is used to kill schistosomes. While schistosome are not sentient, we presume that MgCl_2_ treatment of adults is performed before formaldehyde fixation to quickly kill the individuals before further processing.**

Line 537: was this checked also for Smp_334700?

**Smp_334700 and Smp_186710 were identified as hits containing melibiase-2 domains (PF16499) in our WormBase ParaSite searches. However, both hits conserved less than 70% of this domain and, therefore, did not meet our criterion (defined in the Materials and Methods). We subsequently omitted them from subsequent multiple sequence alignment and phylogenetic analyses. In summary, Smp_186710 and Smp_334700 only contained four or six ligand binding residues, respectively. Both hits also did not contain either catalytic aspartic acid (D) residue (required for NAGAL activity). The alignment below (conserved ligand binding residues = green, conserved catalytic aspartic acid residues = light blue, non-conserved residues in the same positions = red) illustrates this:**

```
Smp_186710      MFTLLTIVSGVVILTLNSVMCLDNGLARTPPMGWNSWLAIDCQVDCVNYPNNCLNENVIK	60
Smp_334700      ------------------------------------------------------------	0
Smp_089290      -------------------------MATVPPMGWMTWQRFRCQTDCQNYPDGCISEKLIK	35


Smp_186710      RTADKLVSDGWRDLGYKYVIIDDCWLSRLRDPKTVRLMPDPSRFPSGMKCLANYLHSKNL	120
Smp_334700      -----------------------------------------------MKNLAQYLHSKNL	13
Smp_089290      RTADRLVLDGWRDLGYRYVIVDDCWPEWKRDSRTNEIVADNKRFPNGIKSVGQYLHSKNL	95
                                                               :* :.:*******

Smp_186710      LFGITIGYGSGTCSGYLGSMDAINICWR--------------------------------	148
Smp_334700      LFGITIGYGTGTCAGYPGSLDFLELDAKTLAEWEVDYVKMNSCNSPDHMMPDGFEKFSRL	73
Smp_089290      RFGIYLDYGTRTCEGYPGSMNYLELDAKSVAKWEADYVKMDGCNSPENIMPDGYGKFSKL	155
                 *** :.**: ** ** **:: :::  :

Smp_186710      ------------------------------------------------------------	148
Smp_334700      LNGTGRPMMFLCTYPLYGSWYAKPELIDWKRLQNNCNLIRALPNSFSSWASVLSIIDGYK	133
Smp_089290      LNATGRPIVFSCSYPAYISWMKNTSLIDWKKLQNNCNLWRMLGDVQDSWSSVISIINAYK	215


Smp_186710      ------------------------------------------------------------	148
Smp_334700      IRNDVLSKVADPGH----------------------------------------------	147
Smp_089290      LQNAVLPKLAGPGHWNDPDMLLMGNFGLSDDQKRVQMGMWCMFSAPLLISADMDKLDNFS	275


Smp_186710      ------------------------------------------------------------	148
Smp_334700      ------------------------------------------------------------	147
Smp_089290      VSLLRNARLLAIDQDKGGHQAEFVKSQNDVQMWVRPLDGDPIGWAIACLFTTDGGGPIHF	335


Smp_186710      ------------------------------------------------------------	148
Smp_334700      ------------------------------------------------------------	147
Smp_089290      YTSLDEFKSQSYRISGDKFELVDVFSGDRFQVVQRTQKFMISINPSGIMMYRVRRVQQKT	395


Smp_186710      -------------	148
Smp_334700      -------------	147
Smp_089290      VSEEIDGLFQTLL	408
```

Fig. S2. The color code is not intuitive. Using blue for males may help to get the point faster.

**The colour code has been changed so male expression values are blue and female expression values are red as requested. The new version of Fig S2 is illustrated below:**

References: italics missing for species names

**All species names within the references list has now been changed to italics as requested.**

**We would like to thank both Reviewers very much for their detailed and very constructive comments, which have helped us to improve our manuscript. We hope that this point-by-point response could clarify the open questions and that our suggested revisions are clear and convincing. We are, of course, happy to discuss any further open issues.**

**Kind regards,**

**Karl F. Hoffmann (on behalf of the authors)**
